# Pomegranate Juice Ameliorates Dopamine Release and Behavioral Deficits in a Rat Model of Parkinson’s Disease

**DOI:** 10.3390/brainsci11091127

**Published:** 2021-08-25

**Authors:** Małgorzata Kujawska, Michael Jourdes, Łukasz Witucki, Marta Karaźniewicz-Łada, Michał Szulc, Agata Górska, Przemysław Ł. Mikołajczak, Pierre-Louis Teissedre, Jadwiga Jodynis-Liebert

**Affiliations:** 1 Department of Toxicology, Poznan University of Medical Sciences, Dojazd 30, 60-631 Poznań, Poland; agorskaaga@gmail.com (A.G.); liebert@ump.edu.pl (J.J.-L.); 2 Institut des Sciences de la Vigne et du Vin, Université de Bordeaux, EA 4577, Œnologie, 210 Chemin de Leysotte, F-33140 Villenave d’Ornon, France; michael.jourdes@u-bordeaux.fr (M.J.); pierre-louis.teissedre@u-bordeaux.fr (P.-L.T.); 3 Institut des Sciences de la Vigne et du Vin, INRA, USC 1366 INRA, IPB, 210 Chemin de Leysotte, F-33140 Villenave d’Ornon, France; 4 Department of Biochemistry and Biotechnology, Poznan University of Life Sciences, Dojazd 11, 60-632 Poznań, Poland; lukwitucki@gmail.com; 5 Department of Physical Pharmacy and Pharmacokinetics, Poznan University of Medical Sciences, Święcickiego 6, 60-781 Poznań, Poland; mkaraz@ump.edu.pl; 6 Department of Pharmacology, Poznan University of Medical Sciences, Rokietnicka 5a, 60-806 Poznań, Poland; mszulc@ump.edu.pl (M.S.); przemmik@ump.edu.pl (P.Ł.M.)

**Keywords:** pomegranate juice, urolithin, ellagitannins, dopamine, olfactory impairment, α-synuclein, non-motor symptoms, motor symptoms, Parkinson’s disease

## Abstract

Pomegranate juice (PJ) is a rich source of ellagitannins (ETs), precursors of colonic metabolite urolithin A, which are believed to contribute to pomegranate’s neuroprotective effect. While many experimental studies involving PJ’s role in Alzheimer’s disease and hypoxic-ischemic brain injury have been conducted, our knowledge of pomegranate’s effects against Parkinson’s disease (PD) is very limited. Previously, we have reported that PJ treatment improved postural stability, which correlated well with enhancement of neuronal survival, protection against oxidative damage, and α-synuclein aggregation. Since olfactory and motor deficits are typical symptoms of PD, in this study, we aimed to investigate the capability of PJ to protect against olfactory, motoric, and neurochemical alterations. To evaluate its efficiency, Wistar rats were given a combined treatment with ROT (1.3 mg/kg b.w./day, s.c.) and PJ (500 mg/kg/day, p.o.) for 35 days. After this, we assessed the olfactory discrimination index (DI) and vertical and horizontal activities as well as levels of dopamine and its main metabolite 3,4-Dihydroxyphenylacetic acid (DOPAC) in the dissected midbrain of animals. Our findings provide the first evidence that PJ treatment protects against ROT-induced DA depletion in the midbrain, which correlates well with improved olfactory function and vertical activity as well as with the presence of urolithin A in the brain.

## 1. Introduction

Parkinson’s disease (PD) is the second most common human neurodegenerative disorder after Alzheimer’s disease (AD). It is a multi-attribute, debilitating condition that leads to significant disabilities and is related to a decreased quality of life over time. The pathological hallmark of PD is intracellular inclusions of misfolded α-synuclein, called Lewy bodies, in the neurons of affected brain regions. Specifically, dopaminergic (DAergic) neurons in the substantia nigra pars compacta (SNpc) undergo degeneration resulting in dopamine (DA) deficiency and multiple other biochemical deficits in the nigrostriatal system [1]. As we previously reviewed, misfolded α-synuclein spreads in a prion-like fashion to different brain regions, giving rise to successive non-motor and motor symptoms [2]. The presence of Lewy bodies in the olfactory bulb and olfactory tract has also been demonstrated [3]. Clinically, PD is characterized by motor dysfunctions such as bradykinesia, rigidity, tremor at rest, postural instability, and non-motor manifestations, including olfactory impairment, pain, autonomic dysfunction, sleep disturbance, fatigue, and behavioral changes [1,3]. Specifically, olfactory impairment precedes the onset of motor symptoms by years [4] and can be used to predict the occurrence of PD in asymptomatic individuals and to differentiate PD from other neurologic disorders [5].

Studies in both a toxin-based [6,7,8] and a transgenic mouse model of PD [9] have presented data suggesting the occurrence of a correlation between the density of nigral DAergic neurons and olfactory discrimination capacity. This association is supported by the findings of Höglinger et al. (2015) demonstrating that there is a direct axonal DAergic projection from the SNpc to the olfactory bulb of rats. The authors suggested that the neurotoxin-induced retrograde degeneration of DAergic neurons in this area could promote the observed hyposmia in rats [10].

In a large case–control study, Belvisi et al. (2020) identified factors inducing PD development: exposure to toxic agents such as pesticides, oils, and metals as well as dyspepsia and general anesthesia. The preventing action has been demonstrated for cigarette smoking, coffee drinking, and physical exercise. No data about the protective effects of medicinal plants have been found in available literature [11].

The pomegranate (*Punica granatum* L.) fruit is rich in various phytochemicals, including ellagitannins (ETs), exerting a wide range of biological activities such as antioxidative, anti-inflammatory, and antiapoptotic activities, which are believed to exert a significant role in its health benefits [12,13]. The neuroprotective effect of pomegranate phytochemicals has been demonstrated against hypoxia-ischemia (H-I) [14,15] and cerebral ischemia-reperfusion (I/R) brain injuries [16]. Regarding neurodegenerative diseases, a lot of in vivo studies on beneficial effects of pomegranate have been devoted to AD [17,18,19,20,21,22,23]; however, its neuroprotective potential against PD is based on very limited data [13,24,25]. We recently suggested that pomegranate’s neuroprotective effect is mediated by urolithin A (UA)—a colonic microbiota ETs-derived metabolite [13]. This is supported by further studies demonstrating the alleviation of cognitive impairments upon treatment with UA in different in vivo models of neurodegeneration [26,27,28].

Previously, we have reported neuroprotective effects of pomegranate juice (PJ) in a rat model of PD based on prolonged low-dose rotenone treatment, which was manifested by improved postural stability correlating well with enhancement of neuronal survival in the SN, protection against oxidative damage, and α-synuclein aggregation in the midbrain. Since olfactory and motor deficits are typical symptoms of PD associated with a decreased DA level, in this study, we aimed to investigate the capability of PJ for counteracting these alterations in the rotenone model of PD and examine whether it is associated with the presence of UA in the brain.

## 2. Materials and Methods

### 2.1. Animals

The experiment was performed on six-week-old male albino Wistar rats (250–300 g) bred at the Department of Toxicology of the Poznan University of Medical Sciences (Poznań, Poland). The animals were held (four rats/cage) in polycarbonate cages (Techniplast, Varese, Italy) with wood shavings in a room maintained under a 12 h light/12 h dark cycle, 22 ± 2 °C, 40–54% relative humidity, and controlled circulation of air. All groups were provided with a commercial diet (ISO 20000 certified laboratory feed Labofeed H) and drinking water ad libitum.

### 2.2. Experimental Design

**Figure 1:**
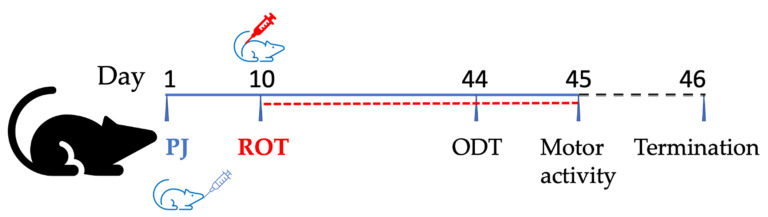
Graphical presentation of the experimental design used in the study.

In order to induce PD in rats, rotenone (ROT, Sigma-Aldrich, Poznań, Poland) was injected subcutaneously once daily for 35 days in a dose of 1.3 mg/kg body weight [13]. Thirty-eight rats were divided randomly into four groups (8 animals in each group plus 3 animals in each of the groups treated with PJ for UA determination in the brain). Group I: rats receiving water (i.g.) and helianthi oleum raffinatum (FAGRON a.s., Olomouc, Czech Republic) (s.c.) from the 11th day, designated as a control group (Control). Group II: rats which were treated only with PJ at a dose of 500 mg/kg b.w./day (i.g.) and injected with helianthi oleum raffinatum from day 11, referred to as the PJ-treated group (PJ). We used commercial 6-fold concentrated pomegranate juice obtained from Alter Medica (Żywiec, Poland) and characterized previously in our laboratory [13]. Group III: rats receiving water (i.g.) and injected with ROT in helianthi oleum raffinatum (1.3 mg/kg b.w./day, s.c.) alone from the 11th day of the experiment, designated as the rotenone group (ROT). Group IV: rats treated with PJ 500 mg/kg b.w./day (i.g.) and injected with ROT from the 11th day, designated as the PJ+ROT group. The experiment lasted 45 days, including 10 days of pre-treatment with PJ and 35 days of combined treatment with PJ and ROT. Twenty-four hours after the last treatment, the rats were anesthetized with ketamine/xylazine (100 U/7.5 mg/kg b.w., intraperitoneally), and blood was withdrawn from the heart (Figure 1). After intracardiac perfusion with isotonic sodium chloride solution, the brain was quickly removed, and the midbrain was dissected on ice, and then snap-frozen with dry ice and stored at −80 °C until further use. For the purpose of UA determination in the brain, whole brains of three rats from groups II and IV were harvested after whole-body perfusion with phosphate-buffered saline, pH 7.4, to avoid overlapping of metabolites from the residual blood.

### 2.3. Behavioural Tests

All tests were carried out between 11:00 and 15:00 and 24 h (motor activity) and 48 h (olfactory discrimination task) before the termination of the experiment in a behavioral testing facility (Figure 1).

#### 2.3.1. Motor Activity

The activity of animals was assessed using an activity cage (40 cm × 40 cm × 31 cm) supplied with infrared beam emitters (Activity Cage 7441, Ugo Basile, Italy) combined with a counter measuring the number of photobeam crossings separately in the horizontal and vertical areas. After previous habituation in the experimental room, the animals were placed in the activity cage, and the examination was performed for 5 min. Software analysis enabled the measurement of the horizontal and vertical activity of the rats.

#### 2.3.2. Olfactory Discrimination Task (ODT)

This test was described by Rodrigues et al. (2014). We used a rectangular box (60 cm × 40 cm × 50 cm) with two interconnected compartments allowing animals free movement. Before testing, the rats were free to explore the apparatus for 5 min. During the test, one compartment was loaded with sawdust with an odor familiar to the animal as it was exposed to the same animals during the preceding 48 h. The second one, endowed with clean sawdust, was designated as a non-familiar odor. After placing a rat in the centrum of the box, the exploratory behavior in the compartments was recorded for 5 min. The animal with olfactory impairment indicating an absence of discrimination tended to explore both compartments equally, while where the olfactory function was intact, the animals preferred to explore a particular compartment [7].

The discrimination index (DI) was calculated by dividing the difference in exploration time between the two compartments (non-familiar − familiar) by the total exploration time for both compartments (non-familiar + familiar). DI was expressed as a percentage, where positive and negative scores correspond to a preference towards non-familiar and familiar odors, respectively. The direction of changes was not considered in the statistical analysis.

### 2.4. Dopamine (DA) and 3,4-Dihydroxyphenylacetic Acid (DOPAC) Level Determination

#### 2.4.1. Isolation

The midbrain tissue was homogenized with an extracting mixture containing acetonitrile–0.1 M HCl–27 mM EDTA water solution (50:40:10) at a weight: volume ratio of 1:10, using a handheld tissue homogenizer and subsequently sonicated for 20 min at 4 °C. Then, the sample was centrifuged at 6500× *g*, and the supernatant was filtered on a 0.2 mm PTFE microfilter before HPLC-MS analysis [29].

#### 2.4.2. UPLC-MS/MS Analysis

The analysis of DA and DOPAC was performed on a Shimadzu Nexera (Shimadzu Co., Kyoto, Japan) chromatograph which contained a five-channel degasser (DGU-20A5) and thermostatted autosampler (SIL-30AC). MS detection was performed on a triple quadrupole mass spectrometer, the LCMS-8030 (Shimadzu Co., Kyoto, Japan). For data processing, the Lab solutions Series Workstation system (Shimadzu, Kyoto, Japan) was applied.

DA and DOPAC were separated in a Gemini^®^ C18 analytical column (150 mm × 2 mm) equipped with a security guard cartridge (Phenomenex, Torrance, CA, USA). The column temperature of 25 °C was maintained by a column oven (Shimadzu^®^ Model CTO-2AC). The mobile phase was a mixture of an aqueous solution of acetic acid of Ph = 2 (A) and methanol (B). The following gradient elution was used: 0–3 min 5% B, 3–5 min linear increase to 70%, 5–8 min 70% B, 8–10 min linear decrease to 5%, 10–12 min 5% B. The mobile phase flow rate was 0.15 mL/min, and the injected sample volume was 20 μL. The eluent from the UPLC column was introduced to the MS detector using electrospray ionization in positive ion mode for the measurement of DA, and in negative ion mode for the measurement of DOPAC. The electrospray needle voltage was 4.5 kV. The desolvation line, the heat block temperature, and the interface temperature were maintained at 250 °C, 400 °C, and 350 °C, respectively. Nitrogen was used as the nebulizing gas and as the drying gas with flow rates of 2 and 10 L/min, respectively. The most sensitive mass transition was from *m*/*z* 154.1 to 136.9 for DA and from *m*/*z* 167.1 to 123 for DOPAC. Linearity of the method was confirmed in the ranges of 0.5–10 ng/mL for DA and DOPAC. The within-run and between-run precision, expressed as relative standard deviations, was <13.7% for DA and <14.8% for DOPAC. The within-run and between-run accuracy of the method, expressed as the relative error, was <14.5%.

### 2.5. Urolithin A Determination

The brains of three rats harvested from each group treated with PJ (group II and IV) were extracted with methanol: HCl (99.9:0.1 *v*/*v*) following enzymatic hydrolysis of conjugated UA metabolites according to Núñez-Sánchez et al. (2014) and Seeram et al. (2006) with some modifications as we described previously [13,30,31]. To assess the concentration of UA in brain homogenates, UPLC-ESI-QTOF-MS analysis was performed, and a calibration curve was established using commercially available UA in the range of 1 ng/mL to 100 ng/mL according to the procedure described previously [13].

### 2.6. Statistical Analyses

The results are presented as mean values ± SEM. Duplicate measurements were carried out, and 8 animals per experimental group were used. For the analysis of UA distribution, 3 animals were used. The control and ROT groups were compared by one-way analysis of variance (ANOVA) followed by Fisher’s LSD test. The threshold for statistical significance was at *p* < 0.05. All statistical analyses and charts were performed using PRISM 8.0 software (GraphPad Software Inc., La Jolla, CA, USA).

## 3. Results

### 3.1. Behavioural Tests

#### 3.1.1. Motor Activity

**Figure 2:**
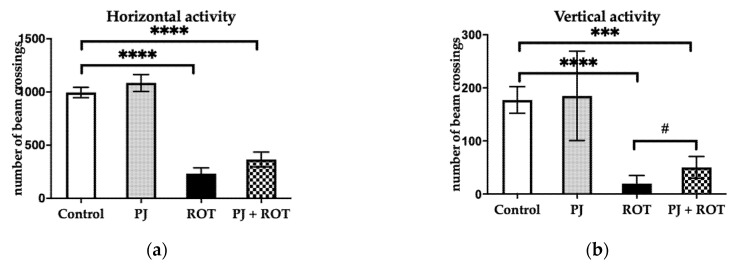
Effect of pomegranate juice treatment (PJ) on: (**a**) horizontal activity; (**b**) vertical activity in rotenone (ROT)-injected rats. Data are presented as mean values ± SEM of eight rats per group and analyzed using one-way analysis of variance (ANOVA) followed by Fisher’s LSD test. **** *p* < 0.0001 vs. Control; *** *p* < 0.001 vs. Control B; # *p* < 0.05 vs. ROT.

Animals injected with rotenone exhibited statistically significant (77% and 89%) lower horizontal and vertical activities, respectively, compared with the Control (Figure 2). Pomegranate juice administration to the ROT-challenged animals attenuated the motor deficit by increasing vertical activity by 160%. A non-significant trend towards increased horizontal activity by 57% was also observed in these animals compared to the ROT group.

#### 3.1.2. Olfactory Discrimination Task (ODT)

**Figure 3:**
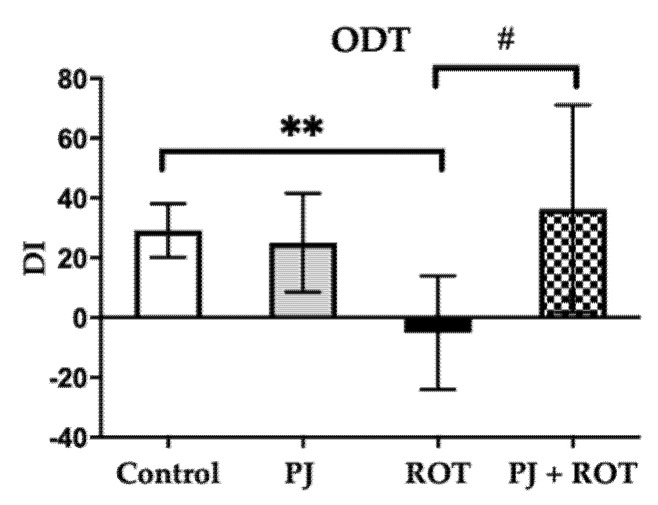
Effect of pomegranate juice treatment (PJ) on olfactory discrimination task (ODT) expressed as olfactory discrimination index (DI) in rotenone (ROT)-injected rats. Data are presented as mean values ± SEM of eight rats per group and analyzed using one-way analysis of variance (ANOVA) followed by Fisher’s LSD test. ** *p* < 0.01 vs. Control. # *p* < 0.05 vs. ROT.

As depicted in Figure 3, rats injected with ROT exhibited impairment of olfactory function attributed to the significantly decreased DI compared to control animals. Treatment with PJ mitigated this ROT-induced effect seen as an increase in DI compared to the ROT group, to the level observed in control animals. Interestingly, the rats receiving PJ and ROT, like the control rats, also showed a preference for the non-familiar odor, inferred from the positive DI scores.

### 3.2. Dopamine (DA) and 3,4-Dihydroxyphenylacetic Acid (DOPAC) Level

**Figure 4:**
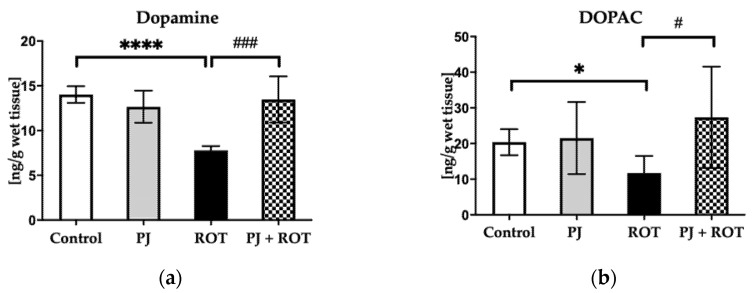
Effect of pomegranate juice treatment (PJ) on: (**a**) dopamine (DA); (**b**) DOPAC levels in the midbrains of rotenone (ROT)-injected rats. Data are presented as mean values ± SEM of eight rats per group and analyzed using one-way analysis of variance (ANOVA) followed by Fisher’s LSD test. **** *p* < 0.0001 vs. Control; * *p* < 0.05 vs. Control, ### *p* < 0.001 vs. ROT; # *p* < 0.05 vs. ROT.

Chronic exposure to ROT reduced dopamine (DA) and its metabolite DOPAC levels by 44% and 43%, respectively, in the midbrain (Figure 4). However, the midbrain DA and DOPAC depletion was significantly attenuated by combined treatment with PJ and ROT, by 73% and 134%, respectively (Figure 4).

### 3.3. Urolithin A Level

The concentrations of UA in the brains of rats treated with PJ alone and in combination with ROT were 2068 ± 0.274 ng/g wet tissue and 0.635 ± 0.174 ng/g wet tissue, respectively, calculated as a mean value ± SD of three rats from the relevant groups.

## 4. Discussion

Although the incidence and prevalence of PD have increased rapidly throughout the world, therapeutic options are still very disappointing, and available therapies treat only the symptoms of the disease. Thus, the optimal management of the neurodegenerative condition is postulated as requiring a multidisciplinary team approach, also encompassing an increasing number of non-pharmacological interventions [32].

The study presented herein is a continuation of the research tackling the question of whether PJ treatment can provide neuroprotection against PD. In rats, long-term exposure to low doses of rotenone, due to sustained inhibition of complex I, related oxidative injury, and α-synuclein aggregation, induced developing degeneration of nigral DAergic neurons with histopathological hallmarks and PD-like symptoms [13,33,34]. DA depletion resulting from a loss of DAergic neurons in the SNpc is considered an important factor underlying the development of motor dysfunction in PD patients [1], which is reproducible in a rotenone PD model [33]. Nigrostriatal denervation is also manifested by a declined level of DOPAC, the main intra-neuronal metabolite of DA [35]. Since we previously observed that PJ offered protection against ROT-induced DAergic neurodegeneration, we therefore anticipated that administration of PJ might thereby mitigate DA depletion and related motor and non-motor deficits. In accordance with previous findings [36,37,38,39], low-dose treatment with ROT induced motor abnormalities manifested in our study, such as decreased horizontal and vertical activities, which were associated with a reduction in DA and DOPAC levels in the midbrain of rats. PJ administration restored the loss of DA level and its metabolite DOPAC, and slightly improved vertical activity. These findings corroborate previous results from Wei et al. (2020), who showed that treatment with ellagic acid (EA) resulted in improvements in the motor performance of rotenone-treated rats [40]. EA also has been reported to protect against 6-hydroxydopamine (6-OHDA) and lipopolysaccharide-induced DA neuronal damage and related motor impairment [41,42,43,44].

Despite the considerable number of studies devoted to the evaluation of neuroprotective effects by assessing motor behavior, very little research involving assessment of olfactory deficits has been conducted in experimental models of PD. Studies from toxin-induced DAergic neuronal loss have shown that accumulation of abnormal α-synuclein is not only confined to the SN but is also found in the olfactory tract [27,45]. Because PJ treatment protected against α-synuclein cumulation in the brain in ROT-challenged rats [13], in this study, we evaluated whether PJ might confer protection against the olfactory deficits. It has been found that ROT injection caused striatal denervation and related DA and DOPAC depletion as well as loss of DAergic neurons in the olfactory bulb that were associated with motor and olfactory impairments [9]. In our study, long-lasting exposure to ROT induced olfactory deficits quantitatively expressed as a decreased DI value. This is consistent with previous work by Rodrigues et al. (2014), who demonstrated the occurrence of a strong association between nigrostriatal DA level and olfactory discrimination capacity [7]. Importantly, PJ treatment prevented the development of PD-like behavioral deficits in ROT-intoxicated rats. EA, a component of PJ, has been reported to alleviate other non-motor symptoms, including hyperalgesia, cognitive deficiency, and memory performance in the 6-OHDA-induced rat model of PD [46,47]. Accumulating evidence supports the beneficial effect of treatment with EA, pomegranate’s natural precursor of UA, against DAergic neuronal loss and related DA depletion and motor and non-motor dysfunctions in PD models [41,42,43,44]. Recently, it has been demonstrated that treatment with extract of *Eclipta alba* rich in EA significantly downregulated the overexpression of α-synuclein at both protein and mRNA levels in the midbrain, prevented loss of DAergic neurons in SNpc, and mitigated behavioral deficits in the MPP+-mediated PD rat model [47].

Our previous findings revealed that UA, the ellagic acid metabolite, is distributed to the brain of PJ-treated rats [13]. In this work, we have demonstrated that PJ treatment prevented the development of PD-like olfactory impairment, slightly mitigated a motor deficit, and preserved DA depletion in ROT-lesioned rats that was accompanied by the presence of UA in their brains. Our findings provide new insights into the beneficial effects of pomegranate juice treatment against PD. Therefore, further studies on plausible mechanisms, including the involvement of UA, that might account for the reported neuroprotective action of pomegranate juice are needed. Since from a neuropathological point of view, both PD and AD are proteinopathies with a long prodromal period characterized by hyposmia [48], it could be suggested that UA may also show potential against impaired olfaction caused by beta-amyloid deposition and a neurofibrillary tangle of the tau formation in the olfactory tract.

## 5. Conclusions

Chronic administration of pomegranate juice prevented dopamine depletion, thereby delaying onset and reducing PD symptoms in rats. Urolithin A, a putative active metabolite formed upon pomegranate juice administration, probably contributed to this effect.

## Data Availability

The datasets analyzed during the current study are available from the corresponding author on reasonable request.
